# Inter-prefectural Travel and Network Connectedness During the COVID-19 Pandemic in Japan

**DOI:** 10.2188/jea.JE20220064

**Published:** 2022-11-05

**Authors:** Cyrus Ghaznavi, Daisuke Yoneoka, Yuta Tanoue, Stuart Gilmour, Takayuki Kawashima, Akifumi Eguchi, Yumi Kawamura, Hiroaki Miyata, Shuhei Nomura

**Affiliations:** 1 Department of Health Policy and Management, School of Medicine, Keio University, Tokyo, Japan; 2 Medical Education Program, Washington University School of Medicine in St Louis, Saint Louis, USA; 3 Infectious Disease Surveillance Center at the National Institute of Infectious Diseases, Tokyo, Japan; 4 Tokyo Foundation for Policy Research, Tokyo, Japan; 5 Institute for Business and Finance, Waseda University, Tokyo, Japan; 6 Graduate School of Public Health, St. Luke’s International University, Tokyo, Japan; 7 Department of Mathematical and Computing Science, Tokyo Institute of Technology, Tokyo, Japan; 8 Center for Preventive Medical Sciences, Chiba University, Chiba, Japan; 9 Department of Global Health Policy, Graduate School of Medicine, The University of Tokyo, Tokyo, Japan

**Keywords:** Japan, human mobility, domestic travel, COVID-19, travel campaign

## Abstract

**Background:**

Increases in human mobility have been linked to rises in novel coronavirus disease 2019 (COVID-19) transmission. The pandemic era in Japan has been characterized by changes in inter-prefectural mobility across state of emergency (SOE) declarations and travel campaigns, but they have yet to be characterized.

**Methods:**

Using Yahoo Japan mobility data extracted from the smartphones of more than 10 million Japanese residents, we calculated the monthly number of inter-prefectural travel instances, stratified by residential prefecture and destination prefecture. We then used this adjacency matrix to calculate two network connectedness metrics, closeness centrality and effective distance, that reliably predict disease transmission.

**Results:**

Inter-prefectural mobility and network connectedness decreased most considerably during the first SOE, but this decrease dampened with each successive SOE. Mobility and network connectedness increased during the Go To Travel campaign. Travel volume between distant prefectures decreased more than travel between prefectures with geographic proximity. Closeness centrality was found to be negatively correlated with the rate of COVID-19 infection across prefectures, with the strength of this association increasing in tandem with the infection rate. Changes in effective distance were more visible among geographically isolated prefectures (Hokkaido and Okinawa) than among metropolitan, central prefectures (Tokyo, Aichi, Osaka, and Fukuoka).

**Conclusion:**

The magnitude of reductions in human mobility decreased with each subsequent state of emergency, consistent with pandemic fatigue. The association between network connectedness and rates of COVID-19 infection remained visible throughout the entirety of the pandemic period, suggesting that inter-prefectural mobility may have contributed to disease spread.

## INTRODUCTION

Depending on local novel coronavirus disease 2019 (COVID-19) infection circumstances, each of Japan’s 47 prefectures enacted up to four statement of emergency (SOE) declarations between 2020 and 2021 at the direction of the central government (eTable 1). During these declarations, Japanese residents were asked to refrain from nonessential activities and movement, restaurants and bars were requested to halt in-person dining services, and employers were encouraged to allow remote work; however, unlike lockdowns enforced in other countries, SOE compliance in Japan was not legally binding.^1^ Despite voluntary compliance, initial SOEs were associated with significant decreases in human mobility,^1^ and the number of individuals staying at home concomitantly increased.^2^ The decrease in domestic travel successfully lowered the risk of COVID-19 importation throughout Japan.^3^ In fact, the relationship between reduced mobility and decreased COVID-19 transmission has been demonstrated in multi-country studies^4^ and in Japan-centric research.^5^^–^^7^ Thus, surveillance of human mobility using mobile phone data during the pandemic has become an integral component of Japan’s COVID-19 containment strategy.^8^

The availability of big mobility data extracted from smartphones has allowed the construction of a significant evidence base detailing the relationship between human movement and COVID-19 transmission, including in Japan.^1^^–^^3^^,^^5^^–^^7^^,^^9^ Mobility is generally characterized as occurring within a defined area (“intra-”) or between areas (“inter”); both types of mobility have been implicated in the spread of COVID-19.^10^^–^^13^ Social distancing measures and travel restrictions are typical policy strategies aimed at reducing these two types of mobility, respectively. In Japan, prior research has found that with each subsequent SOE implementation, compliance with mobility minimization and social distancing measures decreased, likely a manifestation of pandemic fatigue.^9^ However, more than 2 years into the pandemic, it remains unclear how domestic, inter-prefectural travel in Japan changed across SOE declarations and new waves of infection.

After the first SOE, which was implemented in all 47 prefectures, the Japanese government kicked off the Go To Travel campaign (July to December 2020) to boost domestic travel in an attempt to counteract the negative impact of COVID-19 on the tourism industry. The campaign consisted of government-sponsored coupons and discounted travel packages intended to subsidize domestic travel by up to 50%. However, the program was prematurely halted because of increased rates of COVID-19 transmission ostensibly related to the campaign itself,^14^ but tentative plans were made to restart promotional activities in early 2022.^15^ The rise of the Omicron variant has once again jeopardized the return of travel campaigns in 2022.

Given the previously established relationship between domestic travel and COVID-19 importation, as well as future plans for travel campaigns and/or subsequent SOEs, changes in Japanese inter-prefectural movement behavior merit further attention as a public health and policy issue. Here, we use national mobility data taken from smartphone users to assess inter-prefectural travel on a monthly basis during the COVID-19 pandemic in Japan. Furthermore, we quantify network connectedness by tracking changes over time in two network metrics, closeness centrality and effective distance, that have been found to reliably predict the transmission and exportation of infectious diseases.^3^^,^^16^^,^^17^

## METHODS

### Data and calculation of inter-prefectural travel volume

We used Yahoo Japan Corporation data extracted from the smartphone applications of more than ten million users who authorized the use of GPS location data, amounting to more than 10% of Japan’s population. The entire country was divided into 125 meter × 125 meter meshes, and a user was counted as having been in a given area if they remained within the mesh for more than 3 minutes. The mobility data, collected between September 2019 and December 2021, includes the monthly number of unique individuals present in Japan’s 47 prefectures. Each individual’s prefecture of residence is defined independently by Yahoo Japan Corporation based on their nightly location over the past few weeks, which allows the daily number of individuals in each prefecture to be stratified by residential prefecture. Inter-prefectural travel volume was defined as the number of recorded instances of individuals being present in a prefecture that was not their residential prefecture. Thus, the inter-prefectural travel volume was represented by the 47-by-47 adjacency matrix with each element being the travel volume from one prefecture to another; because directionality was considered when calculating travel volume, the adjacency matrix is not symmetric. The number of Yahoo app users who allowed the provision of location information has been extrapolated to the national population (based on population data from the Basic Resident Register) by Yahoo Japan Corporation; we obtained this estimated data.^18^ The data was obtained through an exclusive academic contract between Keio University and Yahoo Japan Cooperation and thus is not publicly available. The number of positive COVID-19 tests and prefecture-level population counts were extracted from online sources.^19^ All data management and calculations were performed in *R* version 4.1.1 (R Foundation for Statistical Computing, Vienna, Austria).

### Calculation of closeness centrality and effective distance

We calculated two network-induced metrics, closeness centrality and effective distance, to assess changes in inter-prefectural travel network structure during the study period. We defined these measures using the associated adjacency matrix of the network.^3^^,^^16^^,^^20^

The closeness centrality, *C_i_*, of the *i*th prefecture in the network is defined as the inverse of the sum of the shortest distance to the other prefectures:

$$C_{i}=\frac{1}{\sum_{j=1,j\neq i}^{46}d_{ji}},$$

where *d_ji_* is the inter-prefectural travel volume on the shortest path from the *j*th to *i*th prefecture. The smaller the *C_i_* value, the larger the number of passengers who travel those paths on the network. The (Pearson) correlation between *C_i_* and the number of positive COVID-19 tests per 1,000 population was calculated. The *igraph* package was used for closeness centrality calculations.

The effective distance, *D_ij_*, between the *i*th and *j*th prefectures is defined as the minimum of all possible effective path lengths (EPL) between the two prefectures.^17^ The *m*th EPL ( $m=1,2,\ldots ,M_{ij}$ ) from the *j*th to the *i*th prefecture with a set of sequence of *l_m_* − 1 transit prefectures, which is denoted by $A_{ij}=\{a_{j},a_{1},\ldots ,a_{l-1}\}$ , is given by

$$L_{m}^{\left(i,j\right)}=l-\log\left(\prod_{k=j,k\in A_{ij}}^{l_{m}-1}P_{k,k+1}\right),$$

where *P_l_*_,_*_m_* denotes the conditional probability that a passenger moved from the *l*th to the *m*th prefecture. The probability was estimated as $P_{l,m}=\frac{d_{lm}}{\sum_{n}d_{ln}}$ , where *d_lm_* is the inter-prefectural travel volume that moved from the *l*th to the *m*th prefecture. Then, the effective distance, *D_ij_*, can be defined as

$$D_{ij}=\min_{\text{m}}L_{m}^{\left(i,j\right)}.$$

In this study, we calculated the effective distances between Aichi, Fukuoka, Hokkaido, Okinawa, Osaka, and Tokyo, where the number of COVID-19 cases were highest in Japan, and each of the remaining 47 prefectures.

## RESULTS

### Inter-prefectural travel volume

**Figure 1.**
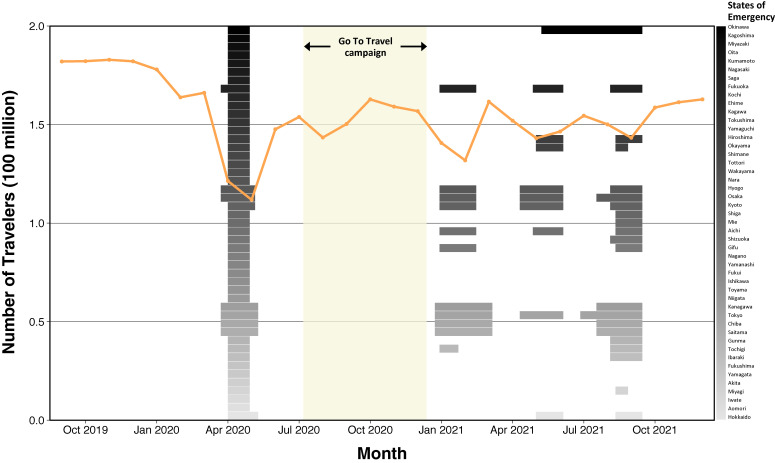
Number of recorded instances of domestic travel between Japanese prefectures per month, September 2019 to December 2021. Rectangles correspond to SOE declaration dates for each of the 47 prefectures (black = Okinawa; light-grey = Hokkaido). Yellow shading corresponds to the Go To Travel campaign period.

The number of individuals travelling outside of their prefecture of residence is shown aggregated by month in Figure 1. In the 4 months leading up to the pandemic (September to December 2019), approximately 180 million instances of people having travelled outside of their residential prefecture were recorded monthly. With the enactment of the first SOE, this number decreased from 166 to 121 million between March and April 2020 and fell further to 112 million in May 2020. The number of travelers rebounded to a peak of 163 million in October 2020 before the second SOE was announced in January 2021, at which point the number decreased to 141 million and reached a trough in February at 132 million. The number of monthly travelers remained between 143 and 163 million (approximately 79–90% of baseline) for the remainder of the analyzed time period, with dips seen around the third and fourth SOEs.

**Figure 2.**
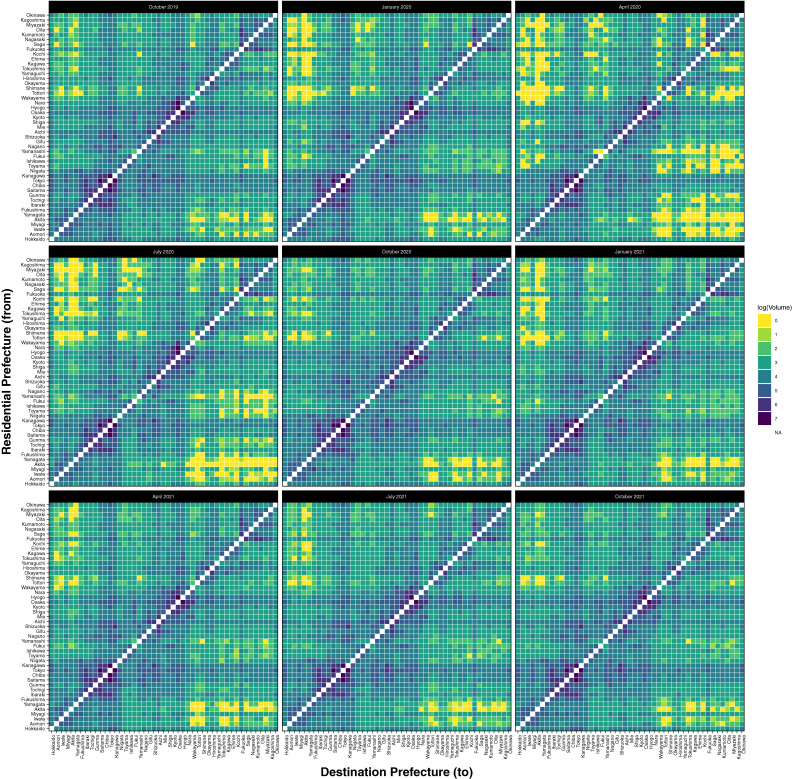
Monthly inter-prefectural travel volume, October 2019 to October 2021. Prefectures are arranged geographically, with Hokkaido being the most northern prefecture and Okinawa being the most southern prefecture. Heatmaps were prepared in R version 4.1.1 using ggplot2. Because the range of values for travel volume between any two prefectures spanned several orders of magnitude, heatmaps display the log-value so as to decrease the range of values to one order of magnitude. These values were rounded to the nearest whole number in order to comply with data privacy protection rules as specified by Yahoo Japan Corporation. For any prefectures with 0 recorded instances of travel to another prefecture, the color corresponds to the lowest value on the color scale.

The network adjacency matrix, showing travel volume between prefectures, is presented as a heatmap, stratified by residence (“from”) and destination (“to”) at the prefectural level, in Figure 2 and eMaterial 1. Prefectures are ordered from south (top-most row, right-most column) to north (bottom-most row, left-most column). Travel volume to nearby prefectures (observed along the diagonal of the heatmap) was several orders of magnitude larger than travel to distant prefectures (top-left and bottom-right of the heatmap). Travel to geographically nearby prefectures remained relatively stable throughout the pandemic, whereas travel to geographically distant prefectures was prone to decreased volumes. Residents of northern (Hokkaido and Tohoku) and southern (Kyushu and Shikoku) Japan exhibited the most striking decrease in travel during April and July 2020 compared to prior months; however, these residents were responsible for low levels of domestic travel compared to the rest of Japan.

### Closeness centrality

**Figure 3.**
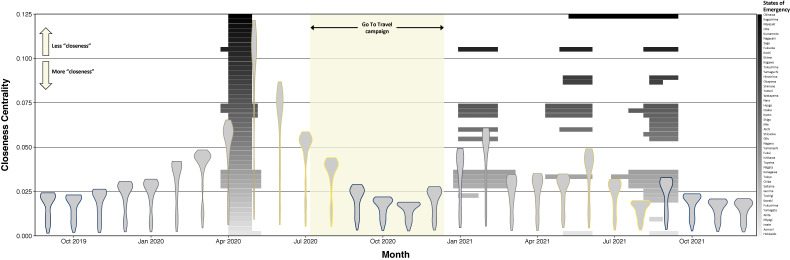
Distribution of closeness centrality by month, September 2019 to December 2021. Each violin plot displays the distribution of closeness centrality scores for all 47 prefectures. The y-axis values are interpreted as distance; thus, larger values indicate less “closeness” with respect to traveler volume. The violin plot itself shows the distribution of closeness centrality scores; thus, the wider the plot, the higher the number of prefectures with the corresponding closeness centrality on the y-axis. Rectangles correspond to SOE declaration dates for each of the 47 prefectures (black = Okinawa; light-grey = Hokkaido). Yellow shading corresponds to the Go To Travel campaign period. Colored outlines surrounding the violin plots correspond to months of the year.

The monthly distribution of closeness centrality across Japan’s 47 prefectures are displayed as violin plots in Figure 3 (see also eMaterial 2); lower values correspond to increased “closeness.” The distribution of closeness centrality shifts upward (ie, less inter-prefectural connectedness) during each of the four SOEs, but the magnitude of the shift decreases with each subsequent SOE. The lower bounds of the distribution, comprised of more metropolitan prefectures, change only slightly compared to the upper bounds. The distribution required approximately 3 months to return to baseline after the first SOE, but subsequent SOEs exhibited more rapid rebounds to normal centrality. The distribution of closeness centrality in October, November, and December 2020 (during the Go To Travel campaign) shifted downward compared to the same months in the previous year. However, the distribution of closeness centrality increased in September 2020 compared to the previous year.

**Figure 4.**
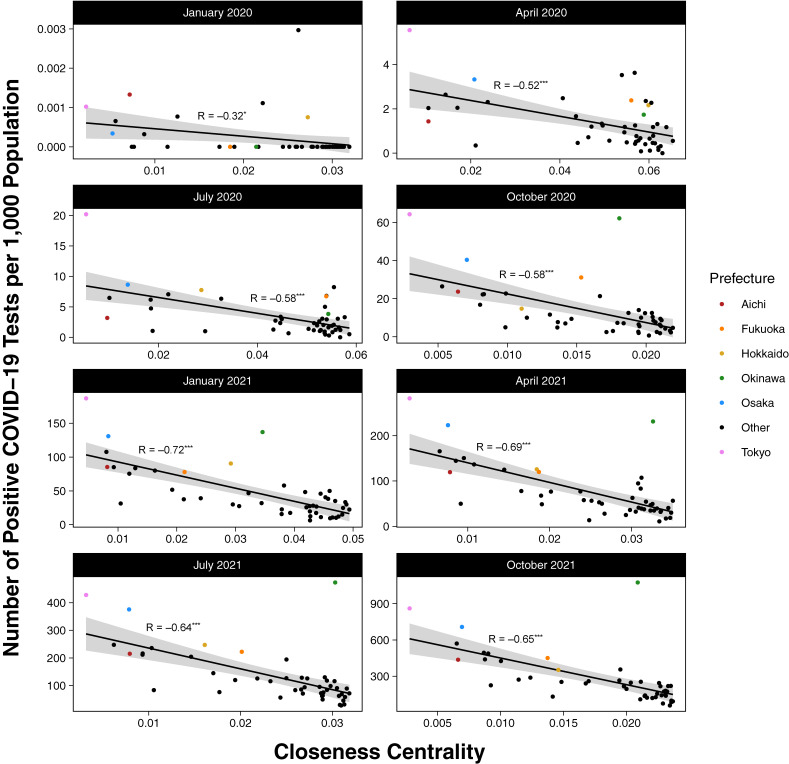
Relationship between closeness centrality and rates of COVID-19 infection by month, January 2020 to October 2021. Each point corresponds to one of Japan’s 47 prefectures. The y-axis values were calculated as the number of positive tests for any given month and prefecture divided by the population of the prefecture (in thousands). Black lines represent lines of best (linear) fit using the ordinary least squares method. Shaded grey areas correspond to standard error of the regression. R indicates the (Pearson) correlation. ^*^, ^**^, and ^***^ denote correlation test *P*-values that are less than or equal to 0.05, 0.01, and 0.001, respectively.

Scatterplots displaying the relationship between closeness centrality and the number of positive COVID-19 cases for any given prefecture are shown in Figure 4 (see also eMaterial 2). For all months shown, the correlation is negative: the lower the closeness centrality, the more positive test results are reported in that same month. The strength of the correlation generally increases as the number of infections rises over time. Six prefectures with the highest rates of COVID-19 infection are illustrated with colored points: Okinawa, Osaka, and Tokyo are consistent outliers with respect to the general, downward, linear trend observed among the remainder of the prefectures.

### Effective distance

**Figure 5.**
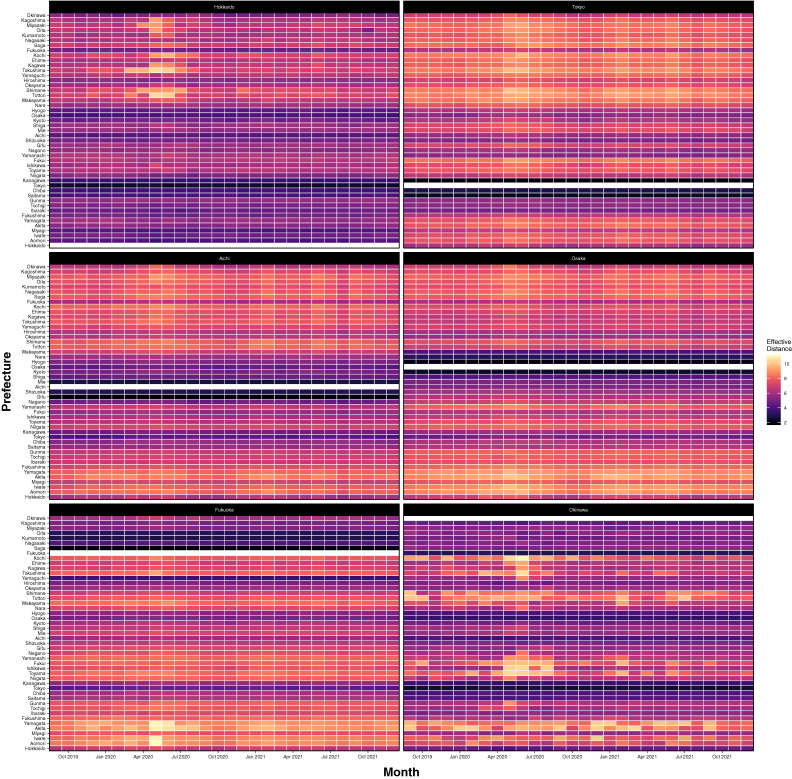
Effective distance between selected prefectures by month, September 2019 to December 2021. Prefectures are arranged geographically, with Hokkaido being the most northern prefecture and Okinawa being the most southern prefecture. Each heatmap displays the effective distance between the prefecture listed in the black striped header of the plot and each of the remaining prefectures listed on the y-axis. Effective distance values are interpreted as distance; thus, larger values indicate being further apart with respect to geography and traveler volume. Heatmaps were prepared in R version 4.1.1 using ggplot2.

The effective distance between Hokkaido, Tokyo, Aichi, Osaka, Fukuoka, and Okinawa and each of the remaining 47 prefectures over time is shown in Figure 5 (see also eMaterial 2); lower values correspond to increased connectivity. The heatmaps for Tokyo, Aichi, and Osaka (all located on the central island of Honshu) suggest that overall, the effective distance with the remaining prefectures did not change considerably during the course of the pandemic, with the main exception being a marginal increase in effective distance recorded during the initial SOEs. Fukuoka, which is located on Kyushu and easily accessible from Honshu, also showed relative stability in its effective distances with the remaining prefectures; however, the effective distance to northern prefectures in Tohoku increased considerably during the first SOE. The effective distance between the northern prefecture of Hokkaido and many southern prefectures increased considerably during the first SOE and marginally during subsequent SOEs. The effective distance between the southern prefecture of Okinawa and several, non-metropolitan prefectures located throughout Japan (eg, Kochi, Tottori, Toyama, and Akita) increased considerably during multiple SOEs.

## DISCUSSION

Using mobility data extracted from the smartphones of more than 10 million Japanese residents, we assessed changes in inter-prefectural travel volume, closeness centrality, and effective distance during the COVID-19 pandemic. We found that the number of travelers decreased during SOEs, but the magnitude of this decrease diminished with each subsequent declaration. When stratified by prefecture, travel to geographically distant destinations decreased more than travel to neighboring prefectures during the pandemic era. Inter-prefectural connectedness was found to have decreased “closeness” during the initial SOEs but were less affected during subsequent declarations. Closeness centrality was found to be negatively correlated with the rate of COVID-19 infections, especially during the mid-to-late phase of the pandemic. Finally, the effective distance between Tokyo, Aichi, Osaka, and Fukuoka and the remaining prefectures was relatively stable over time; however, the effective distance between Hokkaido and Okinawa and smaller prefectures (eg, Kochi, Tottori, Toyama, and Akita) increased considerably during SOEs.

We found that the number of travelers decreased considerably during the first SOE, but subsequent SOEs were associated with smaller and smaller reductions in mobility. This pattern was further corroborated by our analysis of closeness centrality, a measure of inter-prefectural connectedness. These findings are consistent with pandemic fatigue,^21^ which has also been demonstrated in Japan using Google Mobility Index data for general mobility.^9^ As additional rounds of SOE declarations were implemented, efforts to exercise self-restraint became more lax; in fact, there is some evidence to suggest that as vaccination became more widespread in Japan, social distancing decreased.^9^ Notably, we found that decreases in mobility were most noticeable in Northern and Southern Japan, and that travel to neighboring prefectures was largely stable during the pandemic. Research conducted during the early phases of the pandemic found traveler-volume-dependent decreases in COVID-19 infection risk to be most prominent in northern and southern prefectures.^3^ Prior studies have also shown that travel distance decreased in tandem with increases in COVID-19 cases,^22^ and that long-distance trips were more significantly affected than short-distance trips.^23^ That traffic across neighboring prefectures was largely unaffected by SOEs suggests that certain populations may not have altered their behavior in response to voluntary activity restrictions (eg, work commuters and youth travelling from the suburbs to the city). However, the number of monthly travelers only rebounded to 79–90% of baseline in the latter half of the pandemic, consistent with research from the United States that found sustained decreases in travel during the early stages of the pandemic.^24^

When comparing infection rates to closeness centrality among the 47 prefectures over time, we found that increased “closeness” was correlated with higher COVID-19 positive test rates, and that this association became stronger as the number of cases increased. Similar findings have been demonstrated using mobility data in the United States,^24^^,^^25^ Latin America,^26^ and China,^10^^–^^13^ though some studies have found the effects of inter-city travel in China to have limited bearing on infection dynamics.^27^^,^^28^ Notably, the negative correlation between closeness centrality and infection rates remained evident throughout the entirety of the pandemic in Japan, contrasting with prior research that suggests mobility is only a reliable predictor of infection rates during early stages of the pandemic.^26^^,^^29^^,^^30^ Notably, Okinawa, Osaka, and Tokyo were consistent outliers in these analyses. Though outliers did not appear to markedly affect the association between closeness and COVID-19 positivity, the increase in cases nationwide naturally led to increased transmission, especially among those prefectures with the most “closeness.” Osaka and Tokyo are major metropolitan centers with high population density, which also likely contributed to their high rates of infection. Relatively high rates of COVID-19 positivity in Okinawa stand in stark contrast to its isolated geography within Japan but are likely explained by the considerable American military presence that was often associated with infection hotspots.^31^ Okinawa is also a common destination for tourists within Japan, which may have contributed to increased mixing of out-of-prefecture COVID-19-positive individuals and local residents. Of note, the lowest values of closeness centrality (highest “closeness”) were largely observed among the prefectures with the highest populations, which is typically also where the highest rates of COVID-19 were found. We expect that closeness centrality should be lowest in high-population prefectures, as higher populations allow for higher volumes of travel to other prefectures. However, one may also consider that high-population prefectures typically have higher population density and more nightlife/social activities (eg, clubs, bars, or karaoke) than their low-population counterparts, and these factors would contribute to increased COVID-19 transmission.

Effective distance, a metric that combines travel volume with the shortest path between two points, has been shown to be a robust predictor of disease arrival times.^17^ Our analysis of effective distance found that central, metropolitan prefectures such as Tokyo, Aichi, Osaka, and Fukuoka were less likely to be affected during the pandemic and subsequent SOEs than were geographically isolated, less metropolitan prefectures such as Hokkaido and Okinawa. Notably, both Hokkaido and Okinawa are also major tourist destinations, which likely predisposed them to be more sensitive to SOEs and travel campaigns than other prefectures. This is reflected in our analysis of closeness centrality: though the upper bounds of the distributions changed considerably from month to month, the lower bounds (mainly comprised of metropolitan prefectures) were largely unchanged. Notably, research conducted in the United States using mobile phone data found that the relationship between inter-county mobility and COVID-19 infection risk is strongest among the most urban counties.^30^ That travel volumes between urban prefectures were largely sustained during the pandemic suggests that similar trends may have been at play in Japan. Northern and southern prefectures were previously shown to be more significantly impacted by travel restrictions than their central counterparts.^3^ Travel to Hokkaido and Okinawa largely occurs by airplane, whereas travel within the central island of Honshu is split between air travel and ground travel. Limiting domestic airline travel alone has previously been found to be an effective strategy for reducing COVID-19 importation risk,^3^ but research in China has found that ground and rail travel were likely responsible for disseminating disease during the start of the pandemic in Wuhan.^32^ Further research using data stratified by means of travel is necessary to delineate these associations.

The number of monthly travelers rebounded to 90% of baseline during the Go To Travel campaign; this uptick in travel was also reflected in the downward shift of the distribution of closeness centrality between October and December 2020. Indeed, closeness centrality values at the end of 2020 were lower than those in the corresponding period of 2019, suggesting widespread travel that was unusual for the time of year, or more diverse travel patterns. This rapid decrease in closeness centrality was followed in December 2020 by a new, more aggressive wave of infections even though at this time Japan’s borders were tightly controlled. Given our findings that closeness centrality was correlated with monthly infection rates across prefectures, it is possible that the travel campaign led to COVID-19 dissemination, and our findings strengthen the conclusions of prior research that found such an association^14^; however, the current study is unable to make definitive assessments of causality. Further research is needed to evaluate the potential implications of travel campaigns during the pandemic period, as well as identifying the ideal dates of campaign implementation to minimize disease spread.

Our study has limitations. First, if a given smartphone user travels through or stays in more than one prefecture during the same day, that person’s presence will be recorded in multiple prefectures during the same calendar day. Additionally, as we have presented data aggregated by month, many of the same individuals who repeatedly leave their residential prefecture during a given month contributed to multiple instances of recorded travel. Though these values are aggregated, we believe they still adequately capture general trends in inter-prefectural mobility. Second, our data did not include information regarding means of travel (eg, airplane, train, or car) or reason for travel (ie, commuting to work/school vs tourism). In order to design the most efficient and least disruptive travel restrictions, should they be warranted, future studies utilizing this information would be of merit. Third, Yahoo Inc. adjusted the data to reflect the national population with respect to sex and age.^18^ However, adjustments for other variables, such as income, were not performed, as such granular data is not available in this dataset. Fourth, because the incubation period of COVID-19 (generally less than 2 weeks) was shorter than the time-period by which we aggregated data for Figure 4 (monthly), the lag period was absorbed into each month; however, exposures at the end of any given month may have resulted in infections during the next month. Due to data restrictions, we were prohibited from presenting more granular, weekly data in order to take this lag period into account. Finally, we are unable to establish a causal relationship between mobility and COVID-19 infection risk; instead, we note temporal associations between the two phenomena.

## SUPPLEMENTARY MATERIAL

eTable 1. State of emergency declarations by prefecture

eMaterial 1.

eMaterial 2.
