# Supplementary material for: Inter-prefectural Travel and Network Connectedness During the COVID-19 Pandemic in Japan
Source: J Epidemiol. 2022 Nov 5;32(11):510–8. doi: 10.2188/jea.JE20220064 (PMC9551293; doi:10.2188/jea.JE20220064)
Supplement: Supplementary file 1 [file je-32-510-s001.pdf]

eTable 1. State of emergency declarations by prefecture

Prefecture	State of Emergency #1	State of Emergency #2	State of Emergency #3	State of Emergency #4
Hokkaido	4/16/2020 – 5/25/2020	5/16/2021 – 6/20/2021	8/27/2021 – 9/30/2021	
Aomori	4/16/2020 – 5/14/2020			
Iwate	4/16/2020 – 5/14/2020			
Miyagi	4/16/2020 – 5/14/2020	8/27/2021 – 9/12/2021		
Akita	4/16/2020 – 5/14/2020			
Yamagata	4/16/2020 – 5/14/2020			
Fukushima	4/16/2020 – 5/14/2020			
Ibaraki	4/16/2020 – 5/14/2020	8/20/2021 – 9/30/2021		
Tochigi	4/16/2020 – 5/14/2020	1/14/2021 – 2/7/2021	8/20/2021 – 9/30/2021	
Gunma	4/16/2020 – 5/14/2020	8/20/2021 – 9/30/2021		
Saitama	4/7/2020 – 5/25/2020	1/8/2021 – 3/21/2021	8/2/2021 – 9/30/2021	
Chiba	4/7/2020 – 5/25/2020	1/8/2021 – 3/21/2021	8/2/2021 – 9/30/2021	
Tokyo	4/7/2020 – 5/25/2020	1/8/2021 – 3/21/2021	4/25/2021 – 6/20/2021	7/12/2021 – 9/30/2021
Kanagawa	4/7/2020 – 5/25/2020	1/8/2021 – 3/21/2021	8/2/2021 – 9/30/2021	
Niigata	4/16/2020 – 5/14/2020			
Toyama	4/16/2020 – 5/14/2020			
Ishikawa	4/16/2020 – 5/14/2020			
Fukui	4/16/2020 – 5/14/2020			
Yamanashi	4/16/2020 – 5/14/2020			
Nagano	4/16/2020 – 5/14/2020			
Gifu	4/16/2020 – 5/14/2020	1/14/2021 – 2/28/2021	8/27/2021 – 9/30/2021	
Shizuoka	4/16/2020 – 5/14/2020	8/20/2021 – 9/30/2021		
Aichi	4/16/2020 – 5/14/2020	1/14/2021 – 2/28/2021	5/12/2021 – 6/20/2021	8/27/2021 – 9/30/2021
Mie	4/16/2020 – 5/14/2020	8/27/2021 – 9/30/2021		
Shiga	4/16/2020 – 5/14/2020	8/27/2021 – 9/30/2021		

Kyoto	4/16/2020 – 5/21/2020	1/14/2021 – 2/28/2021	4/25/2021 – 6/20/2021	8/20/2021 – 9/30/2021
Osaka	4/7/2020 – 5/21/2020	1/14/2021 – 2/28/2021	4/25/2021 – 6/20/2021	8/02/2021 – 9/30/2021
Hyogo	4/7/2020 – 5/21/2020	1/14/2021 – 2/28/2021	4/25/2021 – 6/20/2021	8/20/2021 – 9/30/2021
Nara	4/16/2020 – 5/14/2020			
Wakayama	4/16/2020 – 5/14/2020			
Tottori	4/16/2020 – 5/14/2020			
Shimane	4/16/2020 – 5/14/2020			
Okayama	4/16/2020 – 5/14/2020	5/16/2021 – 6/20/2021	8/27/2021 – 9/12/2021	
Hiroshima	4/16/2020 – 5/14/2020	5/16/2021 – 6/20/2021	8/27/2021 – 9/30/2021	
Yamaguchi	4/16/2020 – 5/14/2020			
Tokushima	4/16/2020 – 5/14/2020			
Kagawa	4/16/2020 – 5/14/2020			
Ehime	4/16/2020 – 5/14/2020			
Kochi	4/16/2020 – 5/14/2020			
Fukuoka	4/7/2020 – 5/14/2020	1/14/2021 – 2/28/2021	5/12/2021 – 6/20/2021	8/20/2021 – 9/30/2021
Saga	4/16/2020 – 5/14/2020			
Nagasaki	4/16/2020 – 5/14/2020			
Kumamoto	4/16/2020 – 5/14/2020			
Oita	4/16/2020 – 5/14/2020			
Miyazaki	4/16/2020 – 5/14/2020			
Kagoshima	4/16/2020 – 5/14/2020			
Okinawa	4/16/2020 – 5/14/2020	5/23/2021 – 9/30/2021		
